# Strategies to Promote the Integration of Fermented Foods in the Daily Diet to Improve Children’s Health in Capricorn District, South Africa: A Mixed Method Study Protocol

**DOI:** 10.3390/ijerph23070907

**Published:** 2026-07-15

**Authors:** Masilo Isaac Malema, Sogo France Matlala, Paul Kiprono Chelule

**Affiliations:** 1 Department of Public Health, Sefako Makgatho Health Sciences University, 1 Molotlegi Street, Ga-Rankuwa, Pretoria 0208, South Africa; sogo.matlala@smu.ac.za (S.F.M.); paul.chelule@smu.ac.za (P.K.C.); 2 Quality Management Directorate, Walter Sisulu University, Komani Campus, No. 280 Shepstone Road, East London 5200, South Africa

**Keywords:** child nutrition, fermented foods, gut health, mixed methods research, strategy development, traditional diets

## Abstract

**Highlights:**

**Public health relevance—How does this work relate to a public health issue?**

- Diarrhea, malnutrition, stunting, and micronutrient deficiencies remain major contributors to poor child health outcomes in South Africa.
- The study explores the role of fermented foods as a locally available, nutrition sensitive intervention during the critical complementary feeding period.

**Public health significance—Why is this work of significance to public health?**

- It provides evidence on the potential of fermented foods to improve gut health, nutrient absorption, and immunity among children.
- The study addresses gaps in current nutrition programs and guidelines by highlighting underutilized, culturally acceptable dietary practices.

**Public health implications—What are the key implications or messages for practitioners, policy makers, and/or researchers in public health?**

- Findings support and promote the integration of fermented foods into child nutrition policies, guidelines, and community.

**Abstract:**

Child health remains a critical global public health concern, with malnutrition, diarrhea and pneumonia consistently driving high morbidity and mortality rates among children under the age of five. The World Health Organization (WHO) estimates that malnutrition accounts for approximately 3.1 million child deaths annually. In South Africa, persistent challenges such as stunting, micronutrient deficiencies, and food insecurity remain prevalent, particularly in rural communities, despite the existing interventions. Fermented foods present a culturally acceptable, affordable, and sustainable strategy to address malnutrition and improve gut health, potentially reducing stunting and diarrheal diseases. This article presents a protocol for a future four-phase mixed-method study that will be conducted to develop and validate strategies to promote the integration of fermented foods into children’s daily diets. The researchers will employ an exploratory sequential mixed methods design in three phases for the purpose of the envisaged study: bibliometric analysis, qualitative inquiry through in-depth interviews with caregivers and nurses, and a quantitative survey developed from qualitative findings. By combining qualitative and quantitative data, the study seeks to generate evidence-based, context-specific strategies for policy and programmatic integration. The anticipated outcome is a framework to support the inclusion of fermented foods in child nutrition programs to ensure the improved child health and progress toward the United Nations Sustainable Development Goals (UN SDGs).

## 1. Introduction

Child health remains a critical global public health issue, particularly in low- and middle-income countries where malnutrition, diarrhea, and pneumonia continue to be leading causes of morbidity and mortality among children under the age of five. Globally, malnutrition accounts for the deaths of approximately 3.1 million children under the age of five [1]. Addressing this challenge is crucial to achieving the United Nations Sustainable Development Goals (SDGs), especially related to health, nutrition, and food security. In Africa, the burden of child malnutrition is compounded by poverty, food insecurity, and inefficient healthcare systems. More than 25% of children under the age of five are stunted because of chronic malnutrition [2]. While immunization programs and nutrition interventions have yielded measurable improvements, gaps remain in leveraging traditional and culturally relevant dietary practices to improve child health outcomes [3] Under-five mortality remains a major concern in South Africa, with an estimated mortality rate of 35 deaths per 1000 live births [4,5]. Diarrheal diseases, malnutrition, and inequalities in healthcare access persist, particularly in resource-limited provinces such as Limpopo [6]. Stunting affects nearly 29% of children under the age of five, and the nutrition transition characterized by a shift from traditional, nutrient-dense diets to highly processed, energy-dense options has given rise to a dual burden of undernutrition and obesity [7]. Nutrition transition is the gradual shift from traditional, nutrient-rich diets to diets high in processed foods, sugars, and fats, contributing to both undernutrition and childhood obesity.

Fermented foods are integral to many traditional diets across Africa and globally and offer a culturally acceptable entry point for improving nutrition in early childhood. Promoting the consumption of indigenous fermented foods such as fermented cereals and dairy products can support both the cultural preservation of food and improved child nutrition [3,8,9]. Understanding and respecting cultural preferences in food preparation can enhance acceptability and uptake among families and children [10].

Recent studies have highlighted the significant health benefits of fermented foods for children, including improved gut health, enhanced immunity, and potential protection against obesity and the related metabolic disorders [11,12,13,14]. Probiotic-rich fermented foods play a crucial role in shaping gut microbiota, which supports digestion, nutrient absorption, and immune function [15]. In African contexts, products like masi (fermented milk) and munkoyo (fermented cereal beverage) have demonstrated positive impacts on children’s nutrient intake and mineral bioavailability [16].

Emerging evidence also links maternal consumption of fermented food to improved child health outcomes, including sleep duration and immune function, underscoring the intergenerational impact of dietary interventions [17]. Probiotic strains such as *Lactiplantibacillus plantarum* and *Lactobacillus acidophilus* have demonstrated immunomodulatory and nutritional benefits [13,18]. Knowledge gaps and misconceptions among caregivers often limit the use of fermented foods in child diets, despite their proven benefits [19]. Educational programs and food literacy initiatives can improve awareness and increase the consumption of nutrient-rich traditional foods [20,21]. Community based workshops, school programs, and caregiver training have been shown to effectively increase dietary diversity and encourage healthy feeding behaviors [22].

This article outlines the plan for a study to develop evidence-based strategies to integrate fermented foods into the diets of healthy children (0–59 months) [22]. Fermented foods have a long history in African culture, such as masi fermented milk] and munkoyo (cereal-based drinks]. These foods are known to improve mineral bioavailability and gut health [16,23,24]. Despite their benefits, a nutritional transition, characterized by a shift towards Westernized, ultra-processed diets, has led to a significant decline in the consumption of traditional fermented foods among younger caregivers [25,26,27,28]. Traditional fermented foods may offer a culturally appropriate strategy to improve dietary quality and child health during this transition. This transition is often driven by urbanization, the availability of commercial alternatives, and a growing intergenerational gap in the knowledge of traditional preparation techniques [9,29].

While traditional fermented foods are culturally established, there is a significant gap in formal strategies to promote their integration into daily child nutrition at the primary healthcare level. The envisaged study aims to bridge this gap to support long-term health outcomes for healthy children. The study aims to develop evidence-based strategies to promote the integration of fermented foods into child feeding practices. It emphasizes cultural alignment, health promotion, and educational interventions to address persistent gaps in child nutrition and health outcomes. The overarching goal of this research program is to establish a sustainable, evidence-based framework for integrating traditional fermented foods into pediatric nutrition. This protocol serves as the foundational blueprint and specifically focuses on the rigorous development and validation of these strategies before they are piloted in a clinical setting.

The study aims to achieve its goals through the following phased objectives:

- To map the global research trends on fermented foods and child health;
- To explore the knowledge, perceptions, and cultural practices of caregivers regarding fermented foods in children’s diets;
- To explore primary healthcare nurses’ perspectives on the integration of traditional fermented foods into child feeding guidelines;
- To develop context-specific strategies for the integration of fermented foods into the daily diets of children aged between 0 and 5 years in the Capricorn District;
- To validate the proposed strategies.

## 2. Materials and Methods

### 2.1. Research Design

The conceptual landscape and application of mixed methods research (MMR) is often described as the “third methodological movement,” characterized by the strategic integration of qualitative and quantitative elements to provide a comprehensive understanding of complex phenomena [30]. Despite its widespread use, the definition of mixed methods research (MMR) remains a “work in progress,” characterized by evolving terminology and ongoing debates around its conceptual boundaries [31,32]. This diversity is reflected in the use of several related terms, including methodological pluralism, integrated methods, and multi-method research [33,34].

While philosophical and practical challenges to implementation remain, researchers often rely on established design frameworks. One prominent approach is the exploratory sequential design, which is particularly useful for the development of instruments and investigation of under-researched phenomena [35,36]. This design follows a phased process, where initial qualitative findings identify key themes that inform the development and validation of subsequent quantitative instruments [37]. Recent methodological advancements, such as the use of the Delphi technique and Concordance Integration. Typologies further enhance the rigor of this design by systematically examining the alignment between qualitative themes and quantitative metrics [38]. This approach allows for triangulation, strengthening validity and reliability, and providing a comprehensive understanding of the use of fermented foods in child nutrition [39,40].

The study will employ an exploratory sequential mixed-methods design in which qualitative findings will inform the development of the quantitative survey instrument. Integration will occur at three levels. First, qualitative themes emerging from interviews with caregivers and nurses will be used to generate questionnaire items. Second, quantitative findings will be compared with qualitative findings through triangulation and concordance analysis to determine areas of convergence, complementarity, and divergence. Third, integrated findings will inform the development of context-specific strategies for promoting fermented foods in child nutrition. The final strategies will be subjected to expert review and consensus building through a modified Delphi technique. This integration process will enhance the validity, credibility, and applicability of the study findings by combining experiential knowledge with empirical evidence.

### 2.2. Study Roadmap

To ensure a clear progression towards the end goal, the study will follow a sequential transformative design. Phase 1 provides the global scientific context; Phase 2 captures the local socio-cultural reality; Phase 3 synthesizes these data points into actionable nutritional guideline strategies; and Phase 4 subjects these strategies to expert scrutiny.

### 2.3. Study Phases

The study will be conducted in four phases:

- Phase 1: A bibliometric analysis to map research trends on fermented foods and child health.
- Phase 2: A situational analysis through qualitative interviews and quantitative surveys.
- Phase 3: The development of a guideline document with strategies to promote the integration of fermented foods into child diets.
- Phase 4: The final phase of the study will involve the validation of the proposed strategies through stakeholder engagement and expert consensus.

*Expected outputs of each study phase*

Phase 1 Output:

A bibliometric evidence map identifying global research trends, thematic clusters, influential authors, research gaps, and emerging areas related to fermented foods and child health.

Phase 2 Output:

Comprehensive qualitative and quantitative datasets describing knowledge, perceptions, practices, barriers, facilitators, and utilization patterns of fermented foods among caregivers and nurses.

Phase 3 Output:

A draft evidence-informed strategy framework containing practical recommendations for integrating fermented foods into child nutrition programs and household feeding practices.

Phase 4 Output:

A validated consensus-based strategy framework refined through stakeholder engagement and expert review using the modified Delphi technique.

#### 2.3.1. Phase 1: Bibliometric Analysis

**Figure 1:**
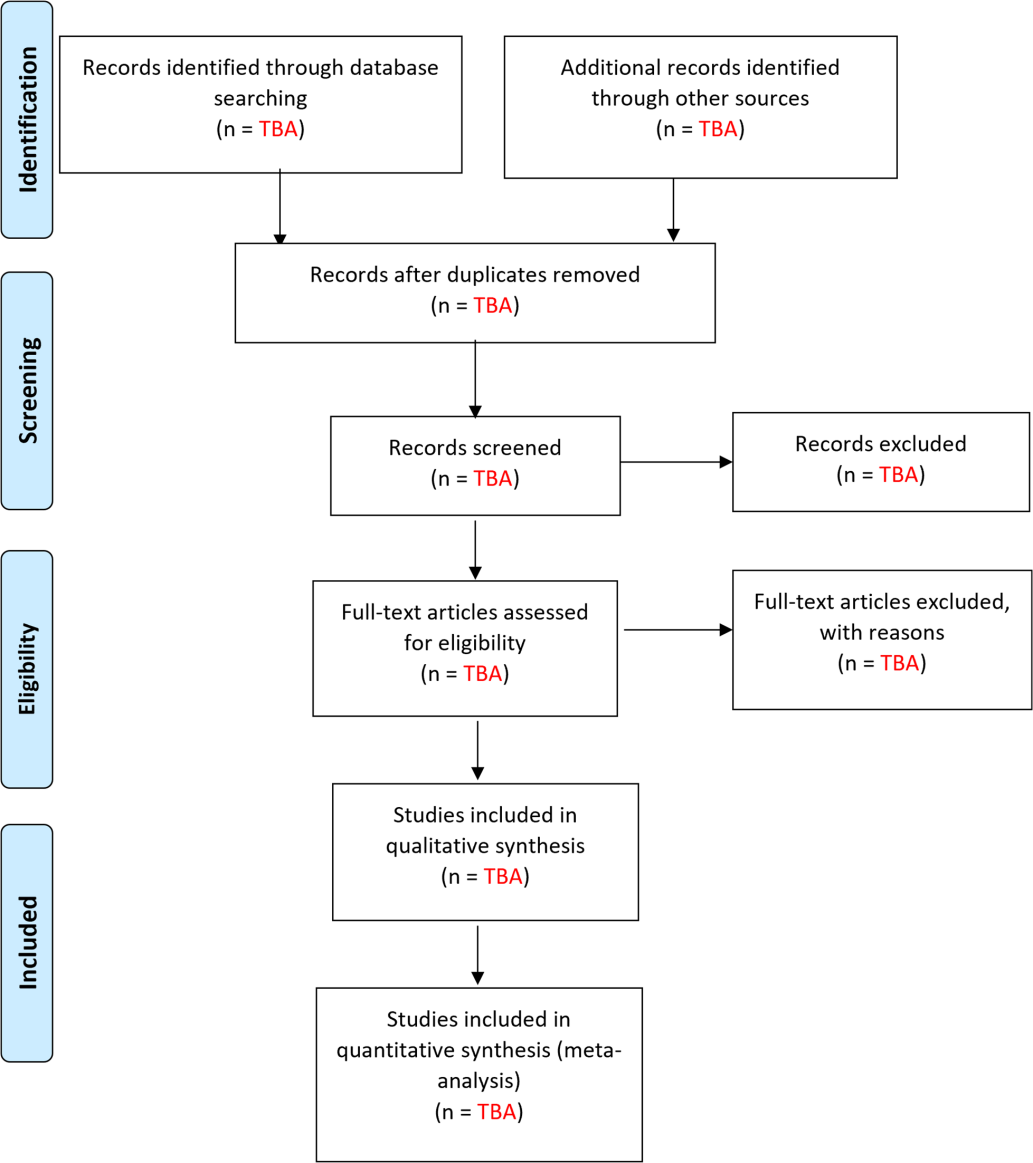
PRISMA flow diagram.

A bibliometric analysis will be conducted to evaluate and synthesize global research on fermented foods and their role in child health. The objective is to map out global research trends on the consumption of fermented foods and child health. The researchers will extract the data from Scopus and the Web of Science, using a Boolean search strategy. The systematic search and selection process will follow the Preferred Reporting Items for Systematic Reviews and Meta-Analyses (PRISMA) guidelines, as shown in Figure 1. Since this is a study protocol, the specific number of records (*n*) at each stage of the identification and screening process is currently marked as ‘TBA’ (to be announced) and will be updated on completion of the analysis [41]; PRISMA is used to ensure transparency in study selection. The choice of bibliometric analysis as the initial phase is justified by its ability to provide a macroscopic and objective evaluation of the scientific landscape of fermented foods and child nutrition. While traditional systematic reviews focus on specific clinical outcomes, bibliometric methods utilize mathematical and statistical modeling to map the intellectual structure of the field. This allows the researchers to identify “hot topics,” emerging trends, and existing research gaps across thousands of publications, information that is crucial for situating the Capricorn District study within the global scientific context. By using software tools like Bibliometrix (R-package), the study will ensure a level of transparency and reproducibility that minimizes researcher bias in literature selection [42,43]. Filters will be applied to include studies published in the last 15 years, focusing on human populations. The Bibliometrix package in R will be used to conduct citation, co-citation, and collaboration network analyses [43]. The bibliometric analysis will identify key research trends, influential authors, thematic clusters, and gaps to guide subsequent phases.

#### 2.3.2. Phase 2: Situational Analysis

##### Study Setting

The study will focus on healthy children under the age of five. Participants will include primary caregivers (mothers, fathers, or guardians) and professional nurses recruited from rural PHC facilities. The study will be conducted in the Capricorn District Municipality, one of the five district municipalities in Limpopo Province, South Africa. The district comprises four local municipalities, namely Blouberg, Lepelle-Nkumpi, Molemole, and Polokwane. According to statistics on South Africa, the district has a predominantly rural population, characterized by high levels of poverty, unemployment, food insecurity, and limited access to specialized health services. Agriculture remains a major economic activity, and traditional food practices continue to play an important role in household food consumption. The selection of a single setting is explicitly justified by the district’s unique public health profile, allowing for a focused, deep-dive situational analysis of rural sub-structures. Consequently, the resulting frameworks are intended primarily for local applicability and context-specific tailoring within the Limpopo Province, rather than broad, un-adapted national generalization.

The Capricorn District was selected because of its high burden of child malnutrition, stunting, diarrheal diseases, and micronutrient deficiencies. Traditional food practices play an important role to affect child health outcomes despite existing nutrition interventions. The district also presents a suitable context for exploring the use of indigenous fermented foods because these foods remain culturally acceptable and locally available. Although the findings will primarily inform interventions within Capricorn District, the resulting strategies may be transferable to similar rural settings in South Africa and other sub-Saharan African countries facing comparable nutritional challenges. The Capricorn District represents a highly critical local setting due to its disproportionately high burden of child stunting (affecting nearly 29% of under-fives) and structural food insecurity.

While the immediate strategies target local implementation within Limpopo Province, the underlying methodology and strategic framework are highly transferable to similar resource-constrained, rural settings across sub-Saharan Africa facing the nutritional transition.

##### Study Population

The study population will comprise caregivers and nurses residing or working within the Capricorn District.

##### Inclusion and Exclusion Criteria

Inclusion criteria

The study will include caregivers who are parents or legal guardians of at least one child aged 0–59 months, who are permanent residents of the selected rural villages and are fluent in either English or Sepedi. In addition, registered nurses working in primary healthcare facilities within the district will be included in the study.

Exclusion criteria

Caregivers of children diagnosed with chronic metabolic disorders requiring specialized medical diets will be excluded from the study. Furthermore, healthcare workers who are not directly involved in maternal and child health (MCH) services will not be eligible to participate.

##### Sampling

The qualitative component will utilize a purposive sample of approximately 30 participants, a sample size considered sufficient to achieve thematic saturation in phenomenological research. For the quantitative component of the study, an automated sample size calculator using Cochran’s parameters (95% confidence level, 5% margin of error, and an assumed 50% distribution prevalence) [44] indicated that a minimum sample size of 383 caregivers and 169 nurses is required. To ensure cognitive suitability and comprehensibility of questionnaire items, cognitive interviewing techniques will be used during pilot testing, guided by the methodological frameworks established by Willis (2005) [45] and the Agency for Healthcare Research and Quality (AHRQ) [46]. The data collection tools will be adapted from validated literature and customized through an expert panel review to ensure content validity. Following the pilot study, construct validity will be statistically evaluated using exploratory factor analysis (EFA), and internal reliability will be verified using Cronbach’s alpha coefficients to ensure structural and scientific robustness before full deployment.

Participants will be asked to explain their interpretation of selected questions, identify unclear terminology, and comment on the appropriateness of response options. Feedback obtained through this process will be used to refine wording, improve question clarity, and minimize measurement error prior to implementation of the main survey.

Separate samples of caregivers and nurses will be utilized to capture a 360-degree view of the nutritional landscape. While caregivers will offer lived experiences of traditional food preparation and child preferences, nurses will provide expert experience regarding the clinical safety and health education barriers within the Limpopo healthcare system.

Stratified random sampling will be used across the four local municipalities within the district to prevent selection bias and ensure proportionate representation of all health facilities.

##### Development of Data Collection Instruments

Qualitative objective: To explore caregivers’ and nurses’ knowledge, perceptions, beliefs, cultural practices, barriers, and facilitators regarding the use of fermented foods in child nutrition. Interview questions will explore participants’ knowledge of fermented foods, perceptions regarding their health benefits, cultural beliefs influencing food choices, current feeding practices, barriers to utilization, facilitators of adoption, and recommendations for integrating fermented foods into child nutrition programs. Example questions include: “What fermented foods are commonly consumed in your household?” “What benefits do you associate with fermented foods?” and “What factors influence your decision to provide fermented foods to children under five years of age?”

The researchers will employ semi-structured interview guides which are available for review as online Supplementary Files (see Annexure A: Interview Guide for Nurses and Annexure B: Interview Guide for Caregivers).

The first (MIM) and second (SFM) authors, who are proficient in both English and Sepedi, will translate the interview guide into Sepedi, the language spoken by the locals in the Capricorn District. To ensure data quality, the researchers will refine instruments to eliminate multi-barreled questions.

Quantitative objective: To quantify the prevalence of fermented food utilization, assess associated factors, and evaluate the relationships between knowledge, attitudes, and practices among caregivers and nurses. The quantitative questionnaire will collect information on demographic characteristics, socio-economic status, knowledge of fermented foods, attitudes towards fermented food consumption, frequency of use, perceived barriers, perceived benefits, and willingness to adopt recommended feeding practices. Responses will be measured using a combination of categorical, multiple-choice, and Likert-scale items.

Quantitative data will be collected through structured questionnaires, developed from the qualitative findings and adapted from validated tools such as the Health Belief Model Scale to evaluate construct domains across both cohorts [47]. The quantitative questionnaire will be developed from themes identified during the qualitative phase and adapted from validated instruments informed by the Health Belief Model. The draft questionnaire will undergo content validation by a multidisciplinary expert panel comprising public health specialists, nutritionists, nurses, and research methodologists. Feedback from the panel will be used to assess relevance, clarity, comprehensiveness, and cultural appropriateness.

A pilot study involving approximately 10% of the calculated sample will be conducted to assess feasibility and comprehension. Construct validity will be assessed using exploratory factor analysis (EFA), while internal consistency reliability will be evaluated using Cronbach’s alpha coefficients. A Cronbach’s alpha value of 0.70 or higher will be considered acceptable [44]. Necessary revisions will be made before implementation of the main survey.

The first (MIM) and second (SFM) authors will translate the questionnaire into Sepedi, using Google translator.

**Summary of the process**:

- **Source**: The tool is newly developed from initial qualitative themes and adapted from the validated *Health Belief Model Scale*.
- **Content Validation**: Conducted by a multidisciplinary expert panel (public health specialists, nutritionists, methodologists).
- **Construct Validity**: Formally evaluated using exploratory factor analysis (EFA) following a 10% pilot study.
- **Reliability**: Evaluated using Cronbach’s alpha coefficients (threshold set at α ≥ 0.70) alongside cognitive interviewing to minimize measurement error.

##### Data Collection Procedure

The researchers obtained ethical clearance [SMUREC/H/219/2025:PG] for the study protocol from the Sefako Makgatho University Research Ethics Committee (SMUREC). Permissions (LP_2025-07-021) to access health facilities and recruit nurses and collect the data from participants in the facilities were obtained from the Limpopo Department of Health (REF: S.5/3/1/2), the office of the Capricorn District Executive Manager, and the PHC facility managers in the Capricorn District. Three tribal authorities gave permission for the researchers to recruit caregivers and collect the data in their villages.

South Africa specifies processes to ensure the facilitation of research and the protection of both participants and institutions. SMUREC requires researchers to obtain and submit permission from authorities before collecting the data. The Limpopo Province Department of Health requires researchers to submit evidence of ethical clearance before granting permission for the research to be conducted, while the district executive manager requires evidence of permission from the provincial office to grant permission to conduct the research. Health facilities require permission from the office of the Capricorn District Executive Manager to allow the researchers to recruit nurses and collect the data from participants in the facilities. Tribal authorities also require evidence of ethical clearance before giving approval for recruitment and data collection. Both the Limpopo Province Department of Health and the Office of Capricorn District Executive Manager require researchers to ensure their studies do not disrupt the routine health services or incur any cost on the department. Furthermore, researchers are required to submit their study findings to the department to serve as a resource and be willing, where possible, to assist with the interpretation and implementation of their study recommendations.

- Recruitment of participants

A multistage recruitment approach will be used. Firstly, villages within the four municipalities will be purposively selected based on population size and accessibility. The recruitment strategy will begin with comprehensive area mapping in collaboration with local community structures to establish a formal sampling frame. Fieldworkers will then systematically utilize a door-to-door household approach methodology within this designated frame to screen and enroll eligible caregivers. The step-by-step workflow utilizes a multistage recruitment approach: initial purposive selection of villages based on population density, structural household mapping via local tribal authorities to construct a localized sampling frame, and an active door-to-door household approach to invite eligible caregivers of children aged 0–59 months. Community leaders and tribal authorities will assist in identifying eligible households. Household mapping will be conducted to establish a sampling frame, after which eligible caregivers of children aged 0–59 months will be approached and invited to participate. The purpose of the study will be explained, and written informed consent will be obtained before participation.

Recruitment of nurses will occur through primary healthcare facilities after obtaining permission from facility managers. Eligible nurses involved in maternal and child health services will be invited to participate voluntarily.

The researchers will conduct interviews with caregivers using either English or Sepedi, and use English only during interviews with the nurses. The MIM is a doctoral candidate who gained research experience when he was reading for his Master’s degree, while SFM and PKC are associate professors with experience in mixed-method research and research supervision. Interview sessions will be guided by interviews guides. These guides will be developed in English and translated into Sepedi. The guides will be semi-structured, with a series of questions that guide the inquiry in line with the objectives. The researchers will use probes to seek clarification on issues arising during the interviews.

##### Data Analysis

The qualitative data will be thematically analyzed using NVivo software (version 15), while quantitative data will be processed using SPSS software (version 30).

Qualitative data analysis

All audio recordings will first be transcribed verbatim by the first author (MIM), using Microsoft 365. The Sepedi audio recordings will be translated into English. Relevant field notes will be added to each transcript to provide contextual insights. The first author (MIM), supervised by SFM and PKC, will conduct a thematic analysis following the six-step framework by Braun and Clarke [48], using NVivo software:

1. *Familiarization*: First, the researchers will individually read through the transcripts to immerse themselves in the data to gain an understanding of the overall content.
2. *Initial coding*: MIM will generate the initial codes from the data, which SFM and PKC will confirm.
3. *Theme development*: The researcher will organize codes into potential themes. This involves grouping similar codes.
4. *Review themes*: The identified themes will be reviewed and refined. This may involve revisiting the data to ensure that the themes accurately represent the content.
5. *Define and name themes*: Each theme will be clearly defined, and given a concise name that reflects its essence.
6. *Reporting*: Last, the findings will be presented, including descriptions of the themes, supporting quotes from the data, and interpretations of their significance.

Trustworthiness will be ensured through credibility, transferability, dependability, and confirmability strategies [49,50].

Quantitative data analysis

The data will be captured in Microsoft Excel, cleaned, and exported to the Statistical Package for the Social Sciences software (SPSS). Descriptive statistics will be computed to summarize demographic data and variables. Chi-square tests will examine associations between variables. Logistic regression will be used to identify predictors of fermented food use among caregivers and nurses.

#### 2.3.3. Phase 3: Strategy Development

The objective of this phase is to co-develop context-specific strategies. Community partners, including pediatricians, registered dietitians, and nutritional experts will be consulted to ensure the strategies meet clinical safety standards and nutritional guidelines. Based on the findings, a participatory emergent strategy will be developed [51]. Interactive stakeholder workshops will inform strategy formulation, while the final strategy will emphasize culturally appropriate, evidence-based integration of fermented foods into child diets.

The application of this strategy will follow these key steps:

1. Stakeholder identification and engagement: MMI will identify and invite key stakeholders such as caregivers, community health workers, nurses, and local leaders to participate in the strategy co-development process.
2. Interactive workshops: Facilitated sessions will be conducted where stakeholders discuss current practices, perceived barriers, and opportunities related to the use of fermented foods in child nutrition.
3. Collaborative strategy formulation: Participants will work together to generate context-specific recommendations, grounded in cultural norms and informed by evidence.
4. Feedback integration and refinement: Emerging strategies will be reviewed and adjusted, based on stakeholder feedback, with a focus on feasibility, cultural relevance, and sustainability.
5. Documentation of the strategy: The final set of strategies will be consolidated into a working proposed strategy template as shown below in Table 1 to guide implementation in community and healthcare settings.

This participatory and flexible approach will ensure that strategies are not only contextually appropriate but are also capable to respond to the dynamic realities of the community. Moreover, the approach will foster a sense of ownership and accountability among stakeholders, increasing the likelihood of successful implementation and sustainability [51]. The proposed strategy template is displayed in Table 1 below.

**Table 1:** Proposed strategy template.

Strategic Objective	Identified Barriers/Needs	Strategic Action/Intervention	Stakeholders Involved	Resources Required	Timeline	Indicators of Success
						

#### 2.3.4. Phase 4: Strategy Validation, Refinement and Consensus

A modified Delphi Technique will be applied to reach consensus on the proposed strategies [52]. The objective of strategy validation is to reach expert consensus on the proposed framework. A modified Delphi Technique will be used, involving 15–20 experts. Expert panel members will include public health specialists, nutritionists, pediatricians, professional nurses, food scientists, and researchers with expertise in child nutrition and fermented foods. Consensus will be defined as at least 70% agreement among participants. Successive Delphi rounds will continue until consensus is achieved or no substantial changes emerge between rounds. The Delphi process will enhance transparency, methodological rigor, and stakeholder ownership of the final strategy framework.

The validation process will involve the following steps:

1. Initial strategy presentation: MIM will present the developed strategies to stakeholders and experts, using an online workshop platform.
2. Structured feedback collection: Participants will be asked to provide feedback on clarity, feasibility, cultural appropriateness, and potential impact through guided discussions and questionnaires.
3. Application of the Delphi Technique: To reach expert consensus, the Delphi Technique will be employed.
4. This structured communication process includes:
  - Round 1: Experts will individually review the proposed strategies and provide feedback.
  - Round 2: A summary of the round one response will be shared with participants, who will be asked to reconsider their views considering group input.
  - Round 3: Final convergence will be sought, highlighting areas of agreement and refining contentious points.
5. Consensus and final refinement: Feedback from all Delphi rounds will be consolidated to modify and finalize the strategies, ensuring they are robust, evidence-based, and community-informed.

## 3. Anticipated Outcomes and Discussion

### 3.1. Anticipated Outcomes

As this document represents a study protocol for a future research implementation, there are no empirical results to report at this stage. The following discussion outlines the conceptual implementation framework, expected baseline contextual factors, and potential scalability pathways based on existing literature. This protocol justifies the use of a mixed-methods approach to ensure that developed strategies are grounded in the specific socio-cultural barriers of the Capricorn District. Unlike clinical trials, this study will focus on implementation evidence to bridge the gap between traditional practices and modern health guidelines. The results of the study will be disseminated to relevant stakeholders; including policy makers, healthcare facility staff, and traditional leaders to support evidence-based decision-making and strengthen community and health system engagement. The findings will further be used to develop a context-specific strategy that aligns with national health priorities and support the reduction of malnutrition, stunting, diarrhea, and other nutrition-related conditions [53,54,55]. The results will also offer practical guidance for policy makers, healthcare providers, and caregivers, contributing to the development of culturally appropriate and sustainable nutrition interventions [3,56].

The successful implementation of the proposed strategies will depend on several contextual factors. Health literacy among caregivers may influence their understanding of the nutritional benefits, preparation methods, and appropriate use of fermented foods in child feeding practices. Furthermore, variations in children’s health and nutritional status may affect the suitability and acceptance of specific fermented food interventions. Food safety considerations, including hygienic preparation, storage practices, and quality control of traditionally fermented products, will be essential to minimize potential health risks and maximize nutritional benefits. These factors will be considered during strategy development and stakeholder consultations to ensure that the final recommendations are both effective and culturally appropriate [57].

### 3.2. Future Directions and Scalability

Following the successful completion of the four phases described in this protocol, the research will transition into an implementation science framework. Future directions include a longitudinal pilot study (Phase 5) to monitor the growth and gut health of children following the integrated diet. This will be followed by a provincial-scale roll-out (Phase 6), in collaboration with the Department of Health. This pathway ensures that the current protocol leads to a tangible improvement in public health outcomes.

Future implementation studies should evaluate not only nutritional outcomes but also caregiver knowledge, health literacy, adherence to recommended feeding practices, and food safety compliance. These factors may significantly influence the sustainability and scalability of fermented food interventions within community and healthcare settings. Capacity-building programs for caregivers and healthcare workers may further support the successful adoption of evidence-informed fermented food strategies.

### 3.3. Study Limitations

This study protocol provides for a robust framework for strategy development; however, it has several limitations. The first limitation is its specific geographic focus on the Capricorn District, which may affect the generalizability of the findings. Second, the qualitative and quantitative data will rely on self-reporting from caregivers and nurses, which may be subject to social desirability bias. Third, the results are reliant on the successful engagement of stakeholders and the accuracy of caregiver-reported utilization frequencies and household feeding patterns, rather than objective nutritional biomarkers or direct dietary intake recalls. As a protocol for a future study, the actual effectiveness of the proposed strategy remains to be evaluated in a subsequent implementation phase.

## 4. Conclusions

This study will provide valuable insights into the role of fermented foods in improving the health and nutritional status of children under the age of five. By employing an exploratory sequential mixed methods design, the research will generate both qualitative and quantitative evidence to support the integration of fermented foods into child nutrition programs. In the long term, this strategy may strengthen public health efforts, enhance child growth and development, and support the achievement of national and global child health targets, including the target set by the SDGs. Ultimately, this protocol seeks to bridge the gap between traditional knowledge and modern clinical nutrition. By providing a clear, four-step path towards strategy validation, we provide a reproducible model for addressing malnutrition and other health conditions in resource-limited settings through the use of locally available, fermented food resources. This research will provide a validated framework for the use of fermented foods as a nutritional intervention. By focusing on healthy children at the village level, these strategies aim to prevent malnutrition before clinical intervention is required.

## Data Availability

No new data have been created for this protocol.

## Supplementary Materials

The following supporting information can be downloaded at: https://www.mdpi.com/article/10.3390/ijerph23070907/s1, Annexure A: Interview guide for Nurses; Annexure B: Interview guides for care givers.

## Abbreviations

The following abbreviations are used in this manuscript:

WHO	World Health Organization
SDGs	Sustainable Development Goals
SMUREC	Sefako Makgatho University Research Ethics Committee
SPSS	Statistical Package for the Social Sciences
