# Supplementary material for: Strategies to Promote the Integration of Fermented Foods in the Daily Diet to Improve Children’s Health in Capricorn District, South Africa: A Mixed Method Study Protocol
Source: Int J Environ Res Public Health. 2026 Jul 15;23(7):907. doi: 10.3390/ijerph23070907 (PMC13411124; doi:10.3390/ijerph23070907)
Supplement: Supplementary file 1 [file ijerph-23-00907-s001.zip › ijerph-4222928-supplementary.pdf]

Annexure A Nurses interview guide

Section A Demographic data

Question A1: What is your age?

Question A2: What is your gender?

Question A3: What is your ethnicity?

Question A4: What is your marital status?

Question A5: What is your level of education?

Question A6: What is employment status?

Question A7: How much is income? (range between … and …)

Question A8: What is their rank in terms of nursing profession?

Question A9: What are your years of experience in nursing?

Section B: Knowledge regarding the benefits of fermented foods

Question B1: Share with me your knowledge of fermented foods.

Question B2: Tell me more about common fermented food? What are they? (Give example of fermented food that you know)

Question B3: Tell us something about how they are prepared. In your household, who prepares fermented food? Where? and how is it prepared? How does culture influence their preparation?

Question B4: Share with me how you integrate fermented foods into your child or children's daily diets or in improving your child's nutrition.

Question B5: What do you think are the health benefits of consuming fermented foods?

Question B6: How do fermented foods contribute to nutrition, improved mental health, improved digestion, improved immune function, etc..?

Question B7: Can you share with me any nutritional advantages of fermentation compared to other food preservation methods?

Question B8: Can you share with me any traditional or medicinal uses or benefits of fermented foods that you are aware of?

Question B9: Do you think fermented foods have economic or environmental benefits? If so, how?

Question B10: How familiar are you with the health benefits of fermented foods for children?

Question B11: What are your thoughts on the suitability of fermented foods for children, and what factors should be considered when including them in a child's diet?

Question B12: Tell me about any cultural beliefs or practices that influence your preparation, acceptance or rejection of fermented foods, particularly for children.

Question B13: Share with me any problems that you face in accessing or obtaining fermented foods in your area.

Question B14: How do the costs of fermented foods affect their usage in your household or practice?

Question B15: What types of educational or awareness programmes would help you better understand and use fermented foods for children?

Question B16: How valuable would workshops or training sessions on fermented food preparation and its benefits be for you?

Question B17: Who do your think should drive these educational and awareness programme on the use of fermented foods?

Question B18: What roles do community support and local (cultural, religious) practices play in the acceptance of fermented foods for child health care?

Question B19: How can community-based initiatives promote the use of fermented foods in child health care?

Section C: Strategies for integrating fermented foods into child health care practices

Question C1: Share with me how you integrate fermented foods into children's daily diets or in improving child's nutrition.

Question C2: What types of educational or awareness programmes would help you better understand and use fermented foods for children?

Question C3: How valuable would workshops or training sessions on fermented food preparation and its benefits be for you?

Question C4: Who do your think should drive these educational and awareness programme on the use of fermented foods?

Question C5: How does the recommendations of fermented foods by healthcare professionals (e.g., nurses, dieticians, doctors, etc.) influence your willingness to use them for children?

Question C6: How might endorsements from healthcare professionals influence the acceptance of fermented foods in your community?

Question C7: What practical steps could facilitate the integration of fermented foods into daily child feeding practices?

Question C8: How can fermented foods be integrated into nutrition policies and guidelines to improve children's health?

Question C9: What factors influence the inclusion of traditional or locally available foods, such as fermented foods, in nutrition policies?

Question C10: How does the Department of Health currently approach the integration of culturally significant foods into dietary guidelines?

Question C11: What role do fermented foods play in child nutrition, and how are they perceived by healthcare professionals and policymakers?

Question C12: What type of research or evidence is needed to support the inclusion of fermented foods in child nutrition guidelines?

Question C13: What challenges could arise in implementing policies or strategy that promote fermented foods for children?

Question C14: How can the Department of Health ensure proper regulation, quality control, and safety of fermented foods in dietary recommendations?

Question C15: What steps can be taken to promote awareness and acceptance of fermented foods among caregivers and communities?

Question C16: What strategies can be used to gain support from key stakeholders, such as policymakers, healthcare professionals, schools (principals), and parents?

Question C17: How can collaboration with local food producers, nutritionists, and traditional health practitioners strengthen the integration of fermented foods into child nutrition programmes?

Question C18: What steps can be taken to advocate for the inclusion of fermented foods in national or provincial nutrition guidelines?

Question C19: What existing pilot projects or community-based initiatives could serve as models for scaling up fermented food i

Annexure B: Caregivers interview guide

Section A Demographic data

Question A1: How are you related to the child? (parent, guardian, caregiver)

Question A2: What is your age?

Question A3: What is your gender?

Question A4: What is your ethnicity?

Question A5: What is your marital status?

Question A6: What is your level of education?

Question A7: What is employment status?

Question A8: How much is income?

Question A9: How many are you in the family?

Question A10: Is the child on social grant?

Question A11: Is your child attending kindergarten/creche or school?

Question A12: How old is the child?

Question A13: What is the gender of the child?

Question A14: Is your have any medical condition?

Question A15: Is your child on any medication?

Section B knowledge regarding the benefits of fermented foods

Question B1: Share with me your knowledge of fermented foods.

Question B2: Tell me more about common fermented food? What are they? (Give example of fermented food that you know)

Question B3: Tell us something about how they are prepared. In your household, who prepares fermented food? Where? and how is it prepared? How does culture influence their preparation?

Question B4: Share with me how you integrate fermented foods into your child or children's daily diets or in improving your child's nutrition.

Question B5: What do you think are the health benefits of consuming fermented foods?

Question B6: How do fermented foods contribute to nutrition, improved mental health, improved digestion, improved immune function, etc..?

Question B7: Can you share with me any nutritional advantages of fermentation compared to other food preservation methods?

Question B8: Can you share with me any traditional or medicinal uses or benefits of fermented foods that you are aware of?

Question B9: Do you think fermented foods have economic or environmental benefits? If so, how?

Question B10: How familiar are you with the health benefits of fermented foods for children?

Question B11: What are your thoughts on the suitability of fermented foods for children, and what factors should be considered when including them in a child's diet?

Question B12: Tell me about any cultural beliefs or practices that influence your preparation, acceptance or rejection of fermented foods, particularly for children.

Question B13: Share with me any problems that you face in accessing or obtaining fermented foods in your area.

Question B14: How do the costs of fermented foods affect their usage in your household or practice?

Question B15: What types of educational or awareness programmes would help you better understand and use fermented foods for children?

Question B16: How valuable would workshops or training sessions on fermented food preparation and its benefits be for you?

Question B17: Who do your think should drive these educational and awareness programme on the use of fermented foods?

Question B18: What roles do community support and local (cultural, religious) practices play in the acceptance of fermented foods for child health care?

Question B19: How can community-based initiatives promote the use of fermented foods in child health care?
